# Spectrum of Complications and Complication Rates After Diagnostic Catheter Angiography in Neuroradiology

**DOI:** 10.1007/s00062-023-01273-3

**Published:** 2023-03-09

**Authors:** Svenja Tafelmeier, Elisabeth Kesseler, Anca-Maria Iancu, Omid Nikoubashman, Martin Wiesmann

**Affiliations:** Department of Diagnostic and Interventional Neuroradiology, University Hospital RWTH Aachen, Pauwelsstr. 30, 52074 Aachen, Germany

**Keywords:** DSA, Stroke, Cerebral angiography, Neurological complications, Technical complications

## Abstract

**Purpose:**

To retrospectively evaluate the total complication rates and type of complications after diagnostic cerebral and spinal catheter angiography.

**Methods:**

Data from 2340 patients undergoing diagnostic angiography over a period of 10 years in a neuroradiologic center were retrospectively evaluated. Local, systemic, neurological, and technical complications were analyzed.

**Results:**

A total of 75 clinically noted complications occurred. The risk for clinical complications was increased when the angiography was performed under emergency conditions (*p* = 0.009). The most common complication was groin hematoma (1.32%). Neurological complications occurred in 0.68% of patients, of which 0.13% were stroke with permanent disability. Technical complications without noticeable clinical symptoms of the patients occurred in 2.35% of the angiographic procedures. Deaths caused by angiography did not occur.

**Conclusion:**

There is a definite risk for complications after diagnostic angiography. Although a very broad spectrum of complications was considered, complications in the individual subgroups showed a low incidence.

## Introduction

Neuroangiography is a common diagnostic tool in the work-up and treatment of neurovascular diseases. It is invasive and therefore associated with a variety of complications [1]. These include local complications at the puncture site [1], technical complications such as failure of transfemoral or radial catheterization [2, 3], as well as embolic stroke [4] and systemic complications such as allergic reactions [1].

Even though there are analyses with large numbers of cases, most of them deal with specific issues only, such as the occurrence of neurological complications [5]. In addition, over the years angiographic materials and techniques have undergone continuous optimization.

Therefore, the aim of our study was to capture a broad range of technical and clinically noted complications and to establish current complication rates in a large cohort of patients undergoing angiography in a specialized neuroradiologic department.

## Material and Methods

### Patients

We retrospectively analyzed imaging and clinical data of all patients who underwent diagnostic neurovascular (cerebral and spinal) neuroangiography at our institution between January 2010 and May 2020. Interventional procedures were not included. We identified a total of 2340 angiographic examinations in 1733 patients. This study was approved by the ethics committee of our institution and has therefore been performed in accordance with the ethical standards laid down in the 1964 Declaration of Helsinki and its later amendments.

### Angiographic Technique

Angiographies were performed on biplanar angiographic systems (Artis Zee, Siemens, Erlangen, Germany). Our standard procedure is as follows: diagnostic angiography is performed by experienced neuroradiologists. Catheterization is achieved via a transfemoral access using 4F or 5F sheaths and respective catheters with angled or sidewinder-configured catheters (Cordis, Santa Clara, CA, USA) using angled-tip standard guide wires (Terumo, Tokyo, Japan). For spinal catheterization, spinal catheters are used (Bentson II Tempo, Spinal Super Torque and Tempo 5 AngioGraphic Catheter C2, Cordis; Soft-Vu Angiographic Catheter Cobra 3, Angiodynamics, Latham, NY, USA; PIG Impress, Merit Medical, South Jordan, UT, USA). Catheters are constantly flushed with a heparinized saline solution (1000 IU heparin diluted in 1000 mL saline) using a high-pressure infusion system. Contrast is achieved with manual injection of a non-ionic contrast medium (Solutrast 300 mg/ml, Bracco Imaging, Konstanz, Germany). Patients are not routinely anticoagulated with heparin and no air filters are used. After angiography, the puncture site is manually compressed for 10–20 min and a pressure dressing applied for 12–24 h, depending on sheath size. In cases with increased hemorrhage risk (dual platelet inhibition, anticoagulation) or when larger sheaths are used, a closure system is applied (Angio-Seal, St. Jude Medical, Minnetonka, MN, USA). All patients are immobilized for at least 12 h and are under clinical observation for at least one night.

### Data Collection and Analysis

For each patient, it was recorded whether diagnostic angiography was performed routinely (e.g., for follow-up), or in the context of an emergency (e.g., for symptomatic internal carotid stenosis). Interventions before and after diagnostic angiography were documented.

Local complications included: hematomas at the puncture site, acute bleeding, thrombosis, and infection.

Technical complications included: difficult puncture, ceasing the angiography, vasospasm, and vascular injury.

Systemic or allergic complications were defined as: headache, nausea, vomiting, transient hypotension, rash, pruritus, sneezing, dyspnea, fever, tremor, paresthesia of the extremities, toxic contrast reaction, confusion and cardiac instability.

Any change in neurological status was recorded. The following neurological symptoms were documented: dysarthria, hemiparesis and hemiplegia, visual disturbances, aphasia, somnolence, weakness of the buccal branch of the facial nerve, and conjugate eye deviation.

Clinical state was classified according to the modified Rankin scale (mRS) at the time of discharge. All neurologic symptoms which normalized within 24 h were defined as transient, within up to 7 days as reversible, and exceeding 7 days as permanent [5]. Patients with reversible and permanent symptoms were followed up by telephone interview as part of our analysis.

### Statistical Analysis

The influence of age, sex and emergency conditions regarding the occurrence of symptomatic, local, systemic, neurological, and technical complications was investigated. Data analysis was performed using SPSS software version 27 (IBM, Armonk, NY, USA). Data distribution was assessed with a Shapiro-Wilk test. Mann-Whitney‑U tests and t‑tests were used for non-parametric and parametric variables, respectively, and χ^2^-tests were used for categorial data. All tests were two-sided and statistical significance (alpha level) was defined as *p* < 0.05.

## Results

A total of 2340 diagnostic catheter angiographies were performed from January 2010 to May 2020 and 1247 (53.29%) angiographies were performed in female patients. The mean age of patients was 55 years (IQR 46–66; range 2 months–94 years), 305 (13.03%) angiographies were performed under emergency conditions, 189 (8.08%) angiographies were spinal, 4F and 5F catheter sheaths were used most frequently (1321/2340; 56.45% and 881/2340; 37.65%, respectively). An angio-seal system was used for puncture site closure in 87 (3.72%) patients.

### Technical Complications

A total of 55 technical complications were noted: these included 24 difficult or failed punctures (1.03%), 12 unfinished examinations (0.51%), 9 vasospasms (0.38%), 9 vascular injuries (0.38%) and 1 iatrogenic arteriovenous (AV) fistula (0.04%) (Table 1).

**Table 1:** Complications in 2340 diagnostic angiographies. Data are presented as *n* (%)

Type of complication	Number of complications (%)
*Local*	
Hematoma	31 (1.32)
*Systemic*	
Headache	11 (0.47)
Nausea and vomiting	5 (0.21)
Transient hypotension	1 (0.04)
Rash	1 (0.04)
Pruritus	1 (0.04)
Fever	1 (0.04)
Toxic contrast medium reaction	2 (0.09)
Cardiac instability	5 (0.21)
Transient confusion	3 (0.13)
*Neurologic*	
Reversible	6 (0.26)
Transient	7 (0.3)
Permanent	3 (0.13)
*Technical*	
Difficult puncture	24 (1.03)
Ceasing the examination	12 (0.51)
Vasospasm	9 (0.38)
Vascular injury	9 (0.38)
Arteriovenous (AV) fistula	1 (0.04)

Of the technical complications 5 occurred after spinal angiography (5/189, 2.65%) and the remaining 50 were noted after cerebral angiography (50/2151, 2.32%). The frequency of technical complications did not differ between spinal and cerebral angiographies (*p* = 0.791).

Puncture was difficult because of scarring (*n* = 2) or atherosclerosis (*n* = 1) and needed to be performed multiple times at the same site in six cases. A switch to the opposite femoral artery side was performed in 13 patients, the brachial artery was used in 2 cases and the radial artery in 1 case. In the remaining 1 case, puncture attempts failed completely.

Angiographies remained unfinished in 12 cases (0.51%), 11 angiographies (0.47%) were aborted because catheterization through the iliac or subclavian level was not possible. Another examination was terminated because the total amount of injected contrast agent was considered too high in a patient with renal insufficiency.

Vasospasm occurred 9 times (0.38%), most frequently in the internal carotid artery (*n* = 6), followed by the vertebral artery (*n* = 1) and segmental spinal arteries (*n* = 1). Nimodipin was administered for vasospasm therapy in 3 of the 9 cases, in all 3 cases with success. In the remaining 6 cases vasospasm normalized without therapy.

Vessel injury in form of dissection occurred 9 times (0.38%), most frequently in the iliac artery (*n* = 5), followed by the internal carotid artery (*n* = 2) and the spinal arteries (*n* = 1). No treatment was needed for these injuries as they were stable and hemodynamically irrelevant.

A small AV fistula (0.04%) occurred after complicated puncture and initial malposition of the sheath in the superficial femoral vein without any consequences.

Age, sex, and emergency conditions had no significant impact on the occurrence of technical complications (*p* = 0.663, *p* = 0.477 and *p* = 0.972, respectively).

### Clinical Complications

A total of 75 complications (3.21%) were noted: 31 were local, 28 were systemic, and 16 were neurological (Table 1).

Of the clinical complications five occurred after spinal angiography (5/189, 2.65%). The remaining 70 clinical complications were observed after cerebral angiography (70/2151, 3.25%). The frequency of clinical complications did not differ significantly between spinal and cerebral angiographies (*p* = 0.690).

The most common local complication was groin hematoma (31/2340; 1.32%). Out of 31 hematomas 2 required treatment (2/2340; 0.09%), in 1 case, because of a hemodynamically relevant hemorrhage that required volume and erythrocyte substitution and in the other case a pseudoaneurysm required surgery. An Angio-Seal system was used in the latter case. Overall, 4 groin hematomas were noted after the use of the Angio-Seal system (4/87, 4.6%), whereas 27 groin hematomas were noted in patients without Angio-Seal (27/2253, 1.2%). The frequency of groin hematomas did not differ significantly with respect to the use of the Angio-Seal system (*p* = 0.066). Taken together, local complications were more likely in patients in whom the angiography was performed under emergency conditions (*p* = 0.042). Age and sex had no significant impact on the occurrence of local complications (*p* = 0.749 and *p* = 0.637, respectively).

The most common systemic complication was headache (11/2340; 0.47%), closely followed by allergic symptoms (9/2340; 0.38%), of which nausea and vomiting were the most frequent. Of the patients five (5/2340, 0.21%) suffered from cardiovascular instability, three of the latter succumbed to a pre-existing cardiovascular disease during subsequent hospitalization but death was not considered to be attributed to angiography. Of the patients three (3/2340, 0.13%) suffered transient confusion and two patients (2/2340, 0.09%) suffered from toxic reactions to the contrast agent. Age, sex, and emergency conditions had no significant impact on the occurrence of systemic complications (*p* = 0.137, *p* = 0.549, and *p* = 0.369, respectively).

Neurological complications (Table 2) during the inpatient stay occurred in 0.68% (*n* = 16) of patients. The majority of complications were transient (*n* = 7, 0.30%) or reversible (*n* = 6, 0.26%). Aphasia and hemiparesis were the most common neurological complications (*n* = 5, 0.21%) followed by dysarthria and visual disturbances (*n* = 4, 0.17%). Facial palsy (*n* = 3, 0.13%), anisocoria (*n* = 2, 0.09%), somnolence (*n* = 2, 0.09%), and conjugate eye deviation (*n* = 1, 0.04%) were less common. No patient suffered from cortical blindness.

**Table 2:** Neurologic complications in 2340 diagnostic angiographies. Data are presented as *n* (%)

Type of Complication	Transient	Reversible	Permanent
Hemiparesis/hemiplegia	0	2 (0.09)	3 (0.13)
Aphasia	1 (0.04)	3 (0.13)	1 (0.04)
Dysarthria	2 (0.09)	1 (0.04)	1 (0.04)
Visual disturbances	2 (0.09)	2 (0.09)	0
Somnolence	1 (0.04)	0	1 (0.04)
Weakness of buccal branch of the facial nerve	1 (0.04)	1 (0.04)	1 (0.04)
Conjugate eye deviation	0	1 (0.04)	0
Anisocoria	1 (0.04)	1 (0.04)	0

Three patients had permanent neurologic symptoms (0.13%): one patient suffered from left-sided hemiparesis with central facial palsy and dysarthria. Another patient suffered from expressive aphasia. The last patient suffered from right-sided hemiparesis.

As shown in Table 3, most of the patients were no longer symptomatic at the time of discharge.

**Table 3:** Classification of neurologic symptoms at the time of discharge

Modified Rankin Scale	No. of patients with transient disability	No. of patients with reversible disability	No. of patients with permanent disability
0	6	5	0
1	0	0	1
2	0	0	0
3	0	2	1
4	0	0	1
5	0	0	0
6	0	0	0

One neurological complication occurred after spinal angiography. After the examination, the patient showed a weakness of the buccal branch of the left facial nerve with dysarthria the next morning. Magnetic resonance imaging (MRI) performed immediately after the angiography showed an acute embolic infarct in the territory of the right middle cerebral artery, as well as small embolic infarcts in the territory of the left middle cerebral artery. Systemic lysis with recombinant human-tissue plasminogen activator (rtPA) was performed. The clinical symptoms regressed completely during the course.

None of the participants with neurological complications died during their inpatient stay. Age, sex, and emergency conditions had no significant impact on the occurrence of neurological complications (*p* = 0.337, *p* = 0.263, and *p* = 0.474, respectively).

Clinically noted complications were recorded in 13 patients (0.56%) who had undergone an angiographic investigation under emergency conditions (13/305, 4.26%). A significant correlation between symptomatic complications and emergency conditions could be found (*p* = 0.009).

Deaths caused by angiography did not occur.

## Discussion

### Technical Complications

To the best of our knowledge, technical complications of diagnostic angiographies, including failed punctures and angiographies, have not been systematically addressed in a larger cohort of patients so far. A main finding of our study is that such technical complications are rare, occurring in approximately 1 out of 50 patients, with difficulties related to arterial puncture being the most frequent complication.

In 24 of our 2340 cases, femoral puncture at the intended side was impossible or considered extremely difficult by an experienced neuroradiologist (1.03%). Of these, puncture was impossible in 16 cases. It needs to be emphasized that for our analysis we used the term “difficult puncture” only for those rare cases when an experienced neuroradiologist considered the difficulties to obtain access so high that this might have increased the patients’ risk for medical sequelae. We are aware, however, that the definition for the term “medical complication” varies throughout the literature. Some authors will consider a case in which femoral puncture was impossible but the angiography could be performed after switching to brachial access (without medical sequelae), not to constitute a complication. For our analysis we have deliberately chosen a different approach. If unforeseen events forced the investigator to deviate significantly from the previously formulated plan (as in the case above) increasing the patients’ risk, although no obvious medical sequelae developed, we considered this a technical complication.

Several authors have investigated the occurrence of iatrogenic dissections with incidences ranging from 0.15% to 0.44% [3, 6–9]. We have found these complications in 0.38% of our patients. While the most common vessel injury in our study was to the iliac artery, the literature indicated injuries to the vertebral [6–9] and internal carotid artery [3, 6] as the most common injuries; however, these studies primarily examined vascular injury and did not examine difficult punctures and catheterization. On the contrary, this may indicate that the frequency of injury to cervical vessels was lower in our cohort.

Olivecrona et al. studied the occurrence of vasospasm [10] and their results are comparable to our results (0.38%). In the study of Thiex et al. a total of 9 technical complications occurred (0.5%). These complications included vessel dissections in 0.2% and sheath-related complications in 0.3% of cases [8]. These results are difficult to compare to our findings. Most of our sheath-related complications have been mentioned under local complications whereas difficult punctures were grouped into the category of technical complications.

Several studies have shown an association between the occurrence of complications and increased age. Especially neurological complications occurred more frequently at an increased age [1, 5, 11, 12]. We did not observe this positive correlation. This could be due to the fact that our total number of cases with neurological symptoms was small. Only 16 patients in our study showed neurological symptoms and 7 of them were under the age of 60 years.

In our study, a correlation between complications, both technical and symptomatic, and sex could not be demonstrated. This is in accordance with the results of Fifi et al. who were also unable to demonstrate a relationship between sex and non-neurological events [12].

### Clinical Complications

Our retrospective review of 2340 diagnostic angiographies revealed a broad range of clinically noted complications.

The incidence of clinically noticed complications associated with diagnostic angiography varies widely in the literature, ranging from 0.3% to 22% [3, 6, 10, 11, 13–16]. This wide range is mostly due to different study designs with varying definitions of complications. For instance, some authors describe a broad spectrum of complications including small groin hematomas [6, 10], whereas others do not include such complications [3]. Taking this into account, our clinical complication rate of 3.21%, including a broad variety of clinically noted complications, may be considered fairly low.

Our most frequent complication was groin hematoma, which accounted for almost half of our complications. Our rate of 1.32% can be considered rather low, as compared to the range of 0.41–10.7% reported in the literature [1, 3, 10–12, 15]. The highest incidences were reported by Olivecrona et al. (10.7%) [10]. Dawkins et al. on the other hand reported lower hematoma rates of 0.41%; however, they used a different definition of groin hematoma and included only hematomas which required additional measures [3]. Under this definition our rate of hematomas requiring treatment was even lower and amounted to 0.09% only.

The complication most feared by both radiologists and patients is stroke. Historically, the risk of a stroke with permanent neurological damage following diagnostic angiography had been estimated to be as high as 1% [17]. Our neurological complication rate of 0.68% is in the lower range of the reported rates of 0.34–2.8% [1, 3, 5, 10–12, 15, 18–20], so is our permanent stroke rate of 0.13%, with the rates reported in the literature ranging from 0.09% to 0.63% [1, 5, 10–16, 18–20]. Both the low rate of neurological complications and the low rate of permanent stroke in our study, may be explained by the experience of the neuroradiologists who performed the angiographies and by the fact that we used protective measures such as constant flushing of catheters with heparinized saline. It may, however, also partly be due to a certain bias, as our analysis was retrospective, and some complications may have been unnoticed. Regarding this, it is noteworthy that we only looked into clinically apparent neurological complications. These are known to be less frequent than clinically silent embolism, which is only visible on MRI and should be subject of further investigations [4].

The overall rate of systemic complications in our study was low, with the most common systemic complication being headache (0.47%). A review of the literature indicated nausea and vomiting as the most common systemic complication. Nausea and vomiting occurred in 0.21% of our patients, which is somewhat lower than rates from the literature. This may be due to the use of modern non-osmotic contrast agents, which are known to have lower complication rates. The more frequent occurrence of headache after angiographies within our study may be due to the different classification of headache into the category of systemic or neurological symptoms. Some authors have defined headache as a neurological sign [10], while in some studies it is not even mentioned as a complication [11, 15].

In accordance with Dawkins et al. we found that emergency conditions were associated with a significantly increased risk of symptomatic complications (*p* = 0.009) [3]. A possible reason for this could be time pressure and the tense situation during an emergency examination, both on the part of the physicians and on the part of the patients, as well as patient’s restlessness and the possible poor medical status.

## Limitations

Due to the retrospective nature of our analysis, a selection bias is possible. Another major limitation is the non-uniform clinical approach. As patients were cared for in both neurological and neurosurgical wards, there was no standard documentation of complications in the discharge report. Documentation was available only up to the time of discharge and not beyond. Complications may have been recorded only if symptomatic. Systematic general or neurologic examinations after angiography were performed only in case of symptoms.

## Conclusion

This study illustrates the occurrence of a broad spectrum of complications of diagnostic cerebral and spinal angiography in a cohort of 2340 patients. Although all types of complications were considered, complications in the individual subgroups showed a low incidence. Most importantly, in our cohort the risk of permanent stroke was 0.13% only. In addition to clinical complications, technical complications also occur. Therefore, a careful case selection and alternative techniques need to be considered.
